# Impact of the use of immunoglobulin palivizumab in the State of São Paulo: a cohort study^1^

**DOI:** 10.1590/1518-8345.1947.2928

**Published:** 2017-09-18

**Authors:** Ivana Regina Gonçalves, Marli Teresinha Cassamassimo Duarte, Helio Rubens de Carvalho Nunes, Rubia de Aguiar Alencar, Cristina Maria Garcia de Lima Parada

**Affiliations:** 2 PhD, RN, Hospital das Clínicas, Faculdade de Medicina, Universidade Estadual Paulista “Júlio de Mesquita Filho”, Botucatu, SP, Brazil. Professor, Faculdades Integradas de Jaú, Jaú, SP, Brasil. Professor Doutor, Faculdade Sudoeste Paulista, Avaré, SP, Brazil.; 3 PhD, Professor, Faculdade de Medicina, Universidade Estadual Paulista “Júlio de Mesquita Filho”, Botucatu, SP, Brazil.; 4 MSc, Statistician, Faculdade de Medicina, Universidade Estadual Paulista “Júlio de Mesquita Filho”, Botucatu, SP, Brazil.; 5 PhD, Adjunct Professor, Faculdade de Medicina , Universidade Estadual Paulista “Júlio de Mesquita Filho”, Botucatu, SP, Brazil.

**Keywords:** Health Policy, Palivizumab, Risk Groups, Newborn, Passive Immunization

## Abstract

**Introduction:**

the use of palivizumab as prophylaxis of the respiratory syncytial virus is not a consensus. In Brazil, it is a public health program, but other countries do not consider it cost-effective.

**Objective:**

to identify the rate of hospitalization in Intensive Care Unit for respiratory illness or symptoms among children who received the immunoglobulin palivizumab, the proportion of children who failed to take any of the recommended doses and the impact of that failure on hospitalization.

**Method:**

cohort study conducted with 693 children enrolled in the palivizumab program in 2014 (85.1% of the population), with monthly assessment from April to September through a telephone call to the mothers or caregiver. The probability of hospitalization in the Intensive Care Unit related to failure in taking the palivizumab, was analyzed through multiple logistic regression, with p<0,05.

**Results:**

the hospitalization rate was 18.2%; 2.3% of the children did not receive all the recommended immunoglobulin doses; the probability of hospitalization for respiratory illness or symptoms increased by an average of 29% at each missed dose (p=0.007; OR=1.29, CI=1.07-1.56).

**Conclusion:**

the increase in the chance of hospitalization related to missed immunoglobulin doses indicates the need to implement health education actions and active search for absent children by the health services.

## Introduction

The use of the immunoglobulin palivizumab for the prophylaxis of the Respiratory Syncytial Virus (RSV) in children under 2 years old is not a worldwide consensus. In some countries, such as Brazil, public health policies^1^ recommend its use for high-risk children, while in others cases it is necessary to establish more restrictive criteria for this measure, due to its low impact on the reduction of hospitalization and of RSV mortality in relation to costs generated^2^. 

Newborns with previous health problems, such as prematurity, heart disease, immunosuppression and Chronic Lung Diseases (CLD), are at higher risk of developing severe illness and requiring hospitalization due to the RSV^3^ ^-^ ^4^. In these cases, it is estimated that, at one year old, 69% of the children will have already shown some symptoms of infection and, at 2 years old, almost all of them will have presented at least one infectious condition and half of them will have had at least two infections^5^. 

Therefore, children with high risk of developing RSV illness should be identified to use this immunoglobulin, since current scientific evidence shows that this group gets the most significant advantages, such as a decrease in the frequency of hospitalization and in morbidity and mortality rates^6^.

Based on data from the Sentinel Surveillance Information System on influenza, the seasonal peak of the virus in the Southeast, Central-West and Northeast regions, was identified as between March and July; in the North Region, this period occurs from February to June; and in the South Region, it occurs from April to August, earlier periods than the ones found in previous assessments^7^.

The first country to approve the use of the immunoglobulin palivizumab in children at high risk of developing RSV infection was the United States of America (USA) in 1998^8^. In the following year its use was also approved in Europe, and then expanded to approximately 50 countries. 

The implementation of the program in Brazil took place in 2013, after the publication of *Portaria* 522, of the Ministry of Health (MH)^1^. The scientific literature on this subject is still scarce, starting from the end of the 1990s. The main existing evidences in the literature are related to the identification and impact of the immunoglobulin in risk groups. Thus, the present investigation is proposed, with the following objectives: to identify the rate of hospitalizations in ICU for respiratory illness or symptoms among children who received the immunoglobulin palivizumab, the proportion of children who failed to receive the correct amount of doses recommended and the impact of this aspect in the hospitalization rates.

## Method

This is a cohort study conducted with children included in the Program for the Use of the Immunoglobulin Palivizumab in the State of São Paulo, conducted during the year 2014, and monitored during the whole period of RSV seasonality (March to August of the same year). 

The legislation with the protocol for the use of palivizumab in Brazil, approved by *Portaria* MH 522 in May 13, 2013, is followed for inclusion in the program in the State of São Paulo^1^.

One of the following criteria must be satisfied for the inclusion of children in the program: premature babies, born with a gestational age of 28 weeks or less and up to one year old or children up to 2 years old with chronic lung disease and/or congenital heart disease with hemodynamic repercussion^1^. Also, the use of the immunoglobulin must start one month before the seasonal period, and be repeated monthly^1^, including during hospitalization.

In 2014, the São Paulo State Department of Health registered 872 children suitable to receive the immunoglobulin palivizumab in one of its 16 application sites. Inclusion criteria for this study were: children up to 19 months old in March 2014 living in the State of São Paulo during the period of seasonality. The exclusion criteria were: death of the mother, identified in the first telephone contact, or of the child during the period of seasonality; difficulties when communicating with the mother; and children living in shelters. The age criteria was defined in order to select children who would not complete 24 months during the seasonal period, which would mean exclusion from the program.

**Figure 1:**
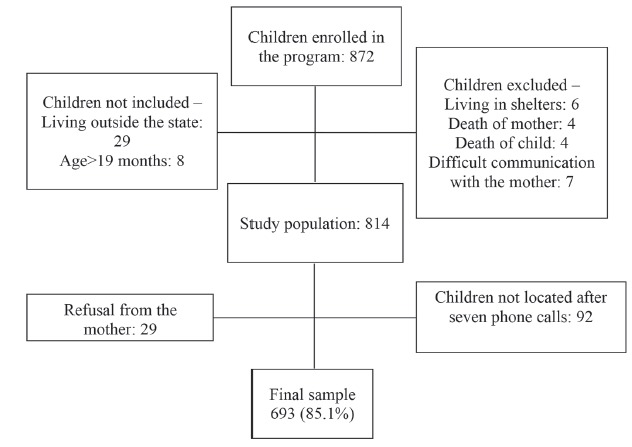
Diagram of the study participants. State of São Paulo, Brazil, 2014

Considering the criteria presented, the study population consisted of 814 children; of these, 121 were excluded (29 mothers refused to participate in the study and 92 were not located after seven telephone calls in at least two different times and days), resulting in a sample of 693 children (85.1% of the population), who composed the cohort of this study. Figure 1, below, synthesizes the composition of the sample.

The variables under study were: application of the immunoglobulin in the Reference Centers for Special Immunobiologic - CRIE (yes, no); variables related to the mother such as color (white, non-white), age in years (up to 19, 20 to 34, 35 or more), schooling in years (up to 10, 11 or more), paid work (yes, no), presence of partner (yes, no) and family income *per capita* (up to 1 minimum wage*, more than 1 minimum wage); variables related to the child, such as gender (male, female), birth weight (grams), history of illness with surgical indication (yes, no), history of clinical illness (yes, no), history of respiratory clinical illness (yes, no), prematurity (yes, no) and gestational age at birth (in complete weeks); and variables related to the household, such as area of living (urban, rural), masonry house (yes, no), number of rooms in the house and number of people sleeping with the child. At the time of data collection, the national minimum wage was R$724,00.

The outcome variable was: hospitalization in the ICU for respiratory illness or symptoms (yes, no), considering the group that followed the protocol and the group that did not. The protocol was considered not followed when there was failure to take one or more doses of the regimen, as recommended by the Ministry of Health^1^.

The exposed group consisted of children who failed to receive at least one of the recommended doses of the immunoglobulin, and the non-exposed group consisted of children who received all doses of the immunoglobulin. 

Subsequent to the cohort of children, data concerning their health situation from March to September 2014 were collected (up to one month after the end of the seasonal period). To that end, the mother or primary caregiver was monthly interviewed by seven trained nurses through a telephone call. On these occasions, the interviewers questioned them about the dates of application of the immunoglobulin, asked if there was a hospitalization in an intensive care unit (ICU) due to respiratory illness or symptoms in the 30 days prior to the telephone call (yes, no), and asked for a description of the evolution of the hospitalization and the child’s current health conditions. 

In the first contact, acceptance to the Consent Form (CF) for Participation in Scientific Studies was recorded in MP3, as were the interviews. Random telephone calls were made by one of the authors to 72 interviewees (approximately 10% of the total number of cases), in order to perform quality control of data collection, and all interviewers were aware of this procedure.

Statistical analysis was carried out through point and interval estimation of the probability of hospitalization in the ICU for respiratory illness or symptoms, in the seasonal period, in two situations: 1) due to at least one failure to receive the immunoglobulin palivizumab in the seasonal period; 2) depending on the number of failures in the seasonal period. In both cases, the analysis of the probability of hospitalization was conducted through multiple logistic regression, based on the most strongly associated variables (p <0.25), identified in the initial bivariate analysis. Relationships were considered statistically significant in the multiple logistic regression if p <0.05, and a 95% CI was used. 

The variables age and schooling of the mother, family income *per capita*, number of people in the room, number of rooms in the house, gestational age and birth weight were treated numerically. The variables application in the CRIE, white mother, mother with 11 years or more of formal education, homemaker mother, presence of partner, area of living, history of surgical pathology, prematurity, history of clinical pathology and history of clinical respiratory pathology were considered as binary. The analysis was performed with the SPSS software, version 21.0.

## Results

Among the 693 children in the cohort sample, 126 (18.2%) were hospitalized for respiratory disease or symptom during the study and 16 (2.7%) failed to receive a dose of the immunoglobulin. Specifically, among the 677 children who took all doses, 123 were hospitalized (18.2%), and among the 16 who failed to receive at least one dose, 3 (18.8%) were hospitalized, with no significant difference between these two groups, p=0.952. 

The characteristics of the mothers and children participating in the study, as well as their living condition, are shown in Table 1.

**Table 1:** Characteristics of mothers and children and their living condition (n=693). State of São Paulo, Brazil, 2014

**Variables**		**N**	**%**
**Color of the mother**			
	**White**	**448**	**64.6**
	**Non White**	**245**	**35.4**
**Age of the mother (years)**			
	**Up to 19**	**40**	**5.8**
	**20 to 34**	**452**	**65.2**
	**35 or more**	**201**	**29.0**
**Schooling (years) of the mother**			
	**Up to 10**	**135**	**19.5**
	**Eleven or more**	**558**	**80.5**
**Mother with paid work**			
	**Yes**	**363**	**52.4**
	**No**	**330**	**47.6**
**Mother with partner**			
	**Yes**	**639**	**92.2**
	**No**	**54**	**7.8**
**Family income** *per capita* in minimum-wages* (in Reais)			
	**Up to 1 minimum wage**	**350**	**50.5**
	**More than 1 minimum wage**	**216**	**31.2**
	**Not known**	**127**	**18.3**
**Gender of the child**			
	**Female**	**346**	**49.9**
	**Male**	**347**	**50.1**
**Child with history of surgical disease**			
	**Yes**	**317**	**45.7**
	**No**	**376**	**54.3**
**Child with history of respiratory clinical illness**			
	**Yes**	**536**	**77.3**
	**No**	**157**	**22.7**
**Weight at birth (grams)**			
	**Up to 1,500**	**448**	**64.7**
	**1,500 to 2,499**	**49**	**7.0**
	**≥2,500**	**147**	**21.3**
	**Not known**	**49**	**7.0**
**Premature child**			
	**Yes**	**582**	**84.0**
	**No**	**111**	**16.0**
**Area of living**			
	**Urban**	**670**	**96.7**
	**Rural**	**23**	**3.3**
**Number of rooms in the household**			
	**1 to 3**	**188**	**27.1**
	**4 to 6**	**454**	**65.5**
**Child sleeps with another person**			
	**Yes**	**536**	**77.3**
	**No**	**157**	**22.7**

*Minimum wage in Brazil: was R$724,00, in 2014.

Regarding the mothers, there was a predominance of white color (64.6%), age between 20 and 34 years (65.2%), 11 years or more of formal education (80.5%), paid work (52.4%), presence of partner (92.2%) and family income *per capita* of up to 1 minimum wage (50.5%). The children were equally divided between genders, 64.7% had a birth weight of less than 1,500 grams, 45.7% had a history of surgical disease, 77.3% had a history of respiratory disease and the premature birth rate was 84.0%. Regarding the living conditions, the majority of the households had between 4 and 6 rooms (65.5%), was located in the urban area (96.7%) and in 77.3% of the cases the child shared the room with another person (Table 1).

All children who received the immunoglobulin lived in a masonry house and had a history of clinical disease. Among premature infants, 53.1% were born with gestational age of 28 weeks or more.

Table 2 is related to the bivariate analysis between variables of interest and the hospitalization of children enrolled in the Program for the Use of Immunoglobulin Palivizumab.

**Table 2:** Hospitalization of children enrolled in the Program for the Use of Immunoglobulin Palivizumab in ICUs, due to respiratory disease or symptoms (n=693), considering the place of application and mother, child and living variables. State of São Paulo, Brazil, 2014

**Variables**		**OR**	**CI 95%**		**p**
Application in CRIE*		**0.76**	**0.52**	**1.11**	**0.158**
**Data on the mother**					
	**White color**	**1.14**	**0.92**	**1.40**	**0.229**
	**Age in years**	**1.00**	**0.97**	**1.03**	**0.864**
	**Education**	**0.95**	**0.81**	**1.11**	**0.526**
	**Eleven years or more of formal education**	**0.68**	**0.43**	**1.06**	**0.087**
	**Homemaker mother**	**1.51**	**1.04**	**2.21**	**0.032**
	**Presence of partner**	**0.67**	**0.36**	**1.26**	**0.216**
	**Family income** *per capita*	**1.00**	**1.00**	**1.00**	**0.101**
**Data on the child**					
	**History of surgical pathology**	**1.35**	**0.93**	**1.98**	**0.116**
	**Prematurity**	**0.92**	**0.59**	**1.46**	**0.735**
	**History of respiratory clinical pathology**	**1.65**	**1.13**	**2.40**	**0.010**
	**Gestational age at birth**	**1.00**	**0.96**	**1.04**	**0.945**
	**Weight at birth**	**1.00**	**1.00**	**1.00**	**0.973**
**Data on the living conditions**					
	**Number of people sleeping with the child**	**1.59**	**1.34**	**1.89**	**0.000**
	**Number of rooms in the household**	**0.94**	**0.83**	**1.06**	**0.308**
	**Household in the urban area**	**1.22**	**0.36**	**4.15**	**0.749**

*Reference Centers for Special Immunobiologic

In the bivariate analysis, the variables that were most associated with hospitalization for respiratory illness or symptoms were: application in CRIE; white mother, with 11 or more years of schooling and working as homemaker; family income *per capita*; number of people sleeping with the child and child with a history of surgical pathology or respiratory clinical pathology (Table 2); therefore, those variables were included in the final model regarding the failure of an application (Table 3) and in the final model that considered the amount of failures in the application of the immunoglobulin palivizumab (Table 4).

**Table 3:** Hospitalization of children enrolled in the Program for the Use of Immunoglobulin Palivizumab in ICUs, due to respiratory illness or symptoms (n=693), considering the occurrence of failure to take at least one dose. State of São Paulo, Brazil, 2014

**Variables**	**OR**	**CI 95%**		**P**
Application in CRIE*	**0.80**	**0.53**	**1.20**	**0.279**
**White mother**	**0.90**	**0.59**	**1.39**	**0.638**
**Mother with 11 years or more of formal education**	**0.68**	**0.41**	**1.13**	**0.137**
**Homemaker mother**	**1.14**	**0.73**	**1.79**	**0.557**
**Family income** *per capita*	**1.00**	**1.00**	**1.00**	**0.871**
**Number of people sleeping in the same room**	**1.25**	**0.99**	**1.57**	**0.059**
**History of surgical pathology**	**1.43**	**0.95**	**2.16**	**0.084**
**History of clinical respiratory pathology**	**1.84**	**1.22**	**2.78**	**0.004**
**Failure to receive at least one dose**	**1.08**	**0.29**	**3.97**	**0.913**

*Reference Centers for Special Immunobiologic

**Table 4:** Hospitalization of children enrolled in the Program for the Use of Immunoglobulin Palivizumab in ICUs, due to respiratory illness or symptoms (n= 693), considering the amount of doses lost. State of São Paulo, Brazil, 2014

**Variables**	**OR**	**CI 95%**		**P**
Application in CRIE*	**0.86**	**0.57**	**1.30**	**0.477**
**White mother**	**0.89**	**0.58**	**1.38**	**0.605**
**Mother with 11 years or more of formal education**	**0.70**	**0.42**	**1.16**	**0.167**
**Homemaker mother**	**1.18**	**0.75**	**1.85**	**0.473**
**Family income** *per capita*	**1**	**1**	**1**	**0.736**
**Number of people sleeping in the same room**	**1.24**	**0.99**	**1.56**	**0.066**
**History of surgical pathology**	**1.43**	**0.95**	**2.16**	**0.089**
**History of clinical respiratory pathology**	**1.95**	**1.28**	**2.95**	**0.002**
**Number of doses missed**	**1.29**	**1.07**	**1.56**	**0.007**

*Reference Centers for Special Immunobiologic

In Table 3, it is possible to observe that the chance of hospitalization in ICU for respiratory illness or symptoms did not differ when comparing children who failed to take one dose to children who took all doses (p=0.913). The variable history of clinical respiratory pathology was independently associated with hospitalization: p=0.004, OR=1.84, CI 95% 1.22-2.78.

The chance of hospitalization in the ICU due to respiratory illness or symptoms was directly proportional to the number of failures. At each dose missed, the chance of hospitalization increased by an average of 29% (p=0.007; OR=1.29, CI 95%=1.07-1.56). In this model, the variable history of clinical respiratory pathology remained independently associated with hospitalization: p=0.002; OR=1.95, CI=1.28-2.95 (Table 4).

## Discussion

This study aimed to identify the impact of failures in the application of immunoglobulin palivizumab on ICU hospitalizations due to respiratory illness or symptom. Failure to receive immunoglobulin at least once during the seasonal period of RSV did not result in an increase in hospitalization. The main finding, however, was the relevance of the number of doses missed, since at each occurrence, the risk of hospitalization increased by an average of 29%. 

A favorable result was also found in a meta-analysis conducted in 2009, which showed a decrease in hospitalization and ICU admission among children who received the immunoglobulin palivizumab when compared to children who received placebo: p=0.0007, relative risk=0.29 and CI 95%=0.14-0.59^9^. The effectiveness of this immunoglobulin was pointed out in other investigations, such as a cohort study carried out in Spain, which identified a hospitalization rate of 13.2% among non-immunized children and of 3.9% among immunized children^10^. Also, a population-based study conducted in two Canadian cities found a reduction in the chance of hospitalization after the implementation of palivizumab (7.3% versus 3.0%)^11^.

In Brazil, a prospective cohort study with 198 children verified that 48 (24.2%) were hospitalized, 30 (15.2%) due to non-respiratory causes and 18 (9.1%) due to respiratory causes. In only one case (0.5%) the RSV^12^ was identified, a value lower than the one found in another study(1.5%)^13^. 

In the present study, the rate of hospitalizations in ICU for respiratory disease or symptom among children receiving all doses was 18.2%, almost twice the value found in the above-mentioned Brazilian cohort^12^. This value was also higher than the ones found in two American studies: The IMpact^8^, a clinical trial that supported the adoption of the palivizumab in the USA, found a hospitalization rate of 4.8%; and a retrospective cohort study conducted between 2003 and 2009 with 8,443 high-risk children found a 7.9% rate of hospitalizations for RSV among children who received all the expected immunoglobulin doses^14)^. 

The explanation for these differences may be the absence of the etiological diagnosis of RSV during the hospitalization of the children. A positive aspect to highlight in this study was the carefulness to avoid memory bias: the questions about hospitalization were asked monthly and only those cases that required hospitalization in the ICU were considered, which, by its severity, would probably not be forgotten by the mother or caregiver in that short space of time.

Another difference can be highlighted: in the IMpact study^8^, the hospitalization rate among children who did not receive all the recommended doses (10.6%) was 55% higher than that observed among the children who received all doses (4,8%). In the present study, the comparison between no failure and one failure resulted in similar rates, respectively 18.2% and 18.8%. A hypothesis that may explain this fact is the proportion of children who failed to receive the immunoglobulin in this study (2.3%), a number significantly lower than that obtained in the IMpact study (7.0%).

The favorable situation of the children who received the immunoglobulin was evident, because, with each dose lost, the chance of hospitalization increased by 29%. Therefore, health services should conduct an active search for the children not receiving the doses according to the immunoglobulin palivizumab protocol^1^ and adopt actions to strengthen the link between health professionals and families, increasing adherence to measures of protection and promotion of child health^15^.

A prospective cohort study conducted with 198 children in the city of São Paulo in 2008 at the Reference Center for Special Immunobiologic of the Federal University of São Paulo^12^ reinforces the need for inclusion of children with chronic lung disease and/or congenital heart disease in the risk group, since they are susceptible to serious infections related to RSV and, consequently, should be indicated to receive the immunoglobulin.

The results of the present study corroborate the need for inclusion of children with a history of respiratory clinical problems in the group that should receive the immunoglobulin during the seasonal period of RSV, since, independently, these children had a 84% greater risk of hospitalization when losing one dose and a 95% greater risk of hospitalization when losing more than one dose. 

These findings are even more relevant due to the fact that lower respiratory diseases are the leading cause of death in children aged 0-5 worldwide, with approximately 906,000 deaths in 2013, corresponding to 14% of all deaths for natural causes in this age group. In Brazil, in the same year and age group, deaths due to lower respiratory tract diseases were also numerically important: 4,255 deaths, 7.9% of the 54,076 deaths due to natural causes^16^.

Regarding more comprehensive studies, a meta-analysis addressing the hospitalization of children aged 2 years or less with bronchopulmonary dysplasia^15^ ^),^ which included prospective and retrospective clinical trials, controlled or not, verified that the weighted mean of hospitalizations among non-treated children was 18.4%, while among children treated with the palivizumab it was 5.6%. It should be noted that these values ​​refer to different samples for the cases treated or not, and that the studies were carried out in different countries^17^.

The non-isolation of the respiratory syncytial virus is a limitation of the present research, since it was not possible to confirm if the hospitalizations were a result of this virus. However, the results highlight the important role of the nurse in the management of the process, including the active search, scheduling the application and education of family members, as well as the active search for the absent patients, in order to reduce hospitalization.

## Conclusion

The use of the immunoglobulin palivizumab among eligible children, as recommended by Brazilian public policies, is important, due to the progressively greater risk of hospitalization in ICU for respiratory illness or symptoms at each dose missed. 

The history of clinical respiratory pathology among children at risk of presenting RSV infection is a factor independently associated with hospitalization in ICU for respiratory illness or symptoms, and therefore, these children need to be carefully monitored.

In general, the importance of not missing immunoglobulin applications was demonstrated. Actions in this direction may include guidance to health professionals to discuss with families the importance of appropriate and frequent applications, implementation of mechanisms for active search of absent patients, and scheduling the applications according to the recommendation.

## Footnotes

* The minimum wage per month in Brazil, nowadays, corresponds to R$ 937,00 *reais* or U$ 283,68 American dollars according to the Central Bank of Brazil on June 28^th^, 2017.
